# Interaction of BES1 and LBD37 transcription factors modulates brassinosteroid-regulated root forging response under low nitrogen in arabidopsis

**DOI:** 10.3389/fpls.2022.998961

**Published:** 2022-09-28

**Authors:** Shuli Chai, Junhua Chen, Xiaolan Yue, Chenlin Li, Qiang Zhang, Víctor Resco de Dios, Yinan Yao, Wenrong Tan

**Affiliations:** ^1^ School of Life Science and Engineering, Southwest University of Science and Technology, Mianyang, China; ^2^ Department of Crop and Forest Sciences & Agrotecnio Center, Universitat de Lleida, Leida, Spain

**Keywords:** brassinosteriods, BES1 transcription factor, LBD37, root forging response, low nitrogen

## Abstract

Brassinosteriod (BR) plays important roles in regulation of plant growth, development and environmental responses. BR signaling regulates multiple biological processes through controlling the activity of BES1/BZR1 regulators. Apart from the roles in the promotion of plant growth, BR is also involved in regulation of the root foraging response under low nitrogen, however how BR signaling regulate this process remains unclear. Here we show that BES1 and LBD37 antagonistically regulate root foraging response under low nitrogen conditions. Both the transcriptional level and dephosphorylated level of BES1, is significant induced by low nitrogen, predominantly in root. Phenotypic analysis showed that *BES1* gain-of-function mutant or *BES1* overexpression transgenic plants exhibits progressive outgrowth of lateral root in response to low nitrogen and BES1 negatively regulates repressors of nitrate signaling pathway and positively regulates several key genes required for NO_3_^-^ uptake and signaling. In contrast, *BES1* knock-down mutant *BES1-RNAi* exhibited a dramatical reduction of lateral root elongation in response to low N. Furthermore, we identified a BES1 interacting protein, LBD37, which is a negative repressor of N availability signals. Our results showed that BES1 can inhibit LBD37 transcriptional repression on N-responsive genes. Our results thus demonstrated that BES1-LBD37 module acts critical nodes to integrate BR signaling and nitrogen signaling to modulate the root forging response at LN condition.

## Introduction

Nitrogen (N) is the main nutrient of plants, its availability is critical for plant growth and crop production (Xu et al., 2012; O’Brien et al., 2016; The et al., 2021). Although the total amount of nitrogen is high in various most ecosystems, available nitrogen is in short supply in agricultural lands, which have resulted in significant limitation for crop growth and yields (O’Brien et al., 2016). Therefore, vast amounts of nitrogen fertilizers have been used in farming to improve the yield of crop (Xu et al., 2012; Vidal et al., 2020; The et al., 2021). However, usage of the large quantites of nitrogen fertilizers not only brings the huge economic costs, but also causes negative effects on ecosystem (Xu et al., 2012; Abascal et al., 2021). Thus, understanding how plants sense, transport, assilimulaiton, and resond to nitrogen is very important. Uncovering the molecular mechanisms invovlved in these processes is critical step for developing crops with N-use efficiency.

To ensure adequate N uptake, plants have developed sophisticated strategies to adapt the fluctuating environmental nutrient availability throught adjusting their physological and morphological responses, such as the induction of nutrient transporters expression at the root tips (Yuan et al., 2007; Wang et al., 2012), the accumulation of metabolites (Fritz et al., 2006; Ren et al., 2021), the changes in root morphology and architecture (Giehl and von Wirén, 2014; Liu et al., 2020) and the excretion of nutrient-mobilizing exudates (Schmid et al., 2014; Canarini et al., 2019). Among these responses, morphological responses of plant root systems are prominet when plant cope with the changing or indequate N abilability (Forde, 2014; Pélissier et al., 2021). Nitrate (NO_3_^-^), one of the main sources of nitrogen in natural and agricultural systems, can serve as the nitrogen signal to modulate plant root system architecture (Forde, 2014; Krapp et al., 2014; Gao et al., 2022). External nitrate has a dural effect on lateral root (LR) growth in *Arabidopsis*. When exposed to high external nitrate concentrations (> 5 mM), lateral root can be developed in nitrate-rich patches but its outgrowth is inhibited by a systemic signal, which is regulated by miR167/ARF8 and miR393/AFB3-mediated auxin signaling (Gifford et al., 2008; Vidal et al., 2010; Hu et al., 2021). Besides this systemic inhibitory effect, both emergence and elongation of lateral root is promoted by mild nitrate deficiency, which is commonly described as nitrogen foraging response (Linkohr et al., 2002; Gruber et al., 2013; Liu et al., 2020). The “systemic foraging strategy” in response to low nitrate is essential for plant fitness and nitrogen acquisition, despite this requires plants to structure their root system at a high cost (Hodge, 2004; O’Brien et al., 2016; Liu et al., 2020). Some molecular components and signaling pathway involved in regulation of the root responses to N/NO_3_^-^ have been identyfied. The simulatory effect of mild nitrate deficiency on lateral root growth is mediated by MADS box transcription factor (TF) ARABIDOPSIS NITRATE REGULATED1 (ANR1) (Zhang and Forde, 1998). ANR1 acts downstream of NITRATE TRANSPORTER1.1 (NRT1.1) and can be activated by NRT1.1, and the activated ANR1 subsequently stimulates lateral root elongation (Remans et al., 2006; Zhang et al., 2019). Furthermore, several phytohoemones appear to play active roles in low N-induced lateral root growth (Vega et al., 2019). In particular, auxin biosynthesis and auxin signaling have been reported to play essential roles in regulation of lateral root growth in response to low N (Vidal et al., 2013; Ma et al., 2014; Hu et al., 2021). Instead, Brassinosteriods (BRs) was also reported to be involved in low-N induced root foraging responses (Jia et al., 2019; Jia et al., 2020; Pandey et al., 2020). However, a lot remains to be discovered about the regulatory mechanisms how low Nitrogen stimulate lateral root growth.

Brassinosteroids (BRs) are a class of plant steroid hormones that play critical roles in numerous aspects of plant growth and development (Nolan et al., 2020). Over the past 30 years, the BR signaling transduction pathway have been well established. BR signaling is perceived by its receptors BRASSINOSTERIOID INSENSITIVE 1 and its co-receptor, BRI1 ASSOCIATED RECEPTOR KINASE 1 (BAK1) (Li and Chory, 1997; Li et al., 2002; Nam and Li, 2002) and then many other signaling components transduct BR signaling in a phosphorylation cascade way to regulate transcriptional factors BRI1 EMS SUPPRESSOR 1/BRASSINAZOLE RESISTANT 1 (BES1/BZR1) in the nucleus to regulate the expression of BR target genes (Wang et al., 2002; Yin et al., 2002; Mora-García et al., 2004; Tang et al., 2008; Kim et al., 2009; Kim et al., 2011). In the absence of BRs, BIN2 phosphorylates BES1 and BZR1, phosphorylted BZR1/BES1 are transported to cytoplasm and thus turn off the BR signaling pathway (Li and Nam, 2002; Youn and Kim, 2015). In the presence of BR, BR binds to its receptors BRI1 and BAK1 through activating their transphosphorylation, the activated BRI1 then activates downstram BR SIGNALING KINASEs (BSKs), CONSTITUTIVE DIFFERENTIAL GROWTH 1 (CDG1), and BRI1-SUPPRESSOR 1 (BSU1), and thus leading to inactivation of BIN2 kinase (Guo et al., 2013). BIN2 inactivation results in the location of nonphosphorylated form of BES1 and BZR1 in nucleus to regulate BR target genes (Yin et al., 2002; He et al., 2005; Wang et al., 2021).

BR is invovled in regulation of plant root reponses to nutrient deficiency such as nitrogen and phosphorus. In Arabidopsis, the BR signaling gain-of-function mutant, *bzr1-1D* exhibited insensitive to the low Pi condition in its roots. Low Pi treatment leads to a reduction in BR biosynthesis and a decrease in BES1/BZR1 nucleus localization that ultimately inhibits BR-mediated root growth (Singh et al., 2014). Instead, BRs positively regulate the root forging response at nitrogen deficiency in *Arabidopsis*. Natural genetic variation screening among the *Arabidopsis thaliana* accessions showed that natural variations of brassinosteroid (BR) signaling kinase *BSK3* leads to variation of its activity which contributes to the root foraging response under low N (Jia et al., 2019). In addition, *DWARF 1* (*DWF1*), a key gene involved in BR biosynthesis, was also found to modulate root elongation under low nitrogen. Mild nitrogen deficiency can activate BR biosynthesis though upregulates *DWF1* expression and thus stimulate root elongation under this condition (Jia et al., 2020). Consistent with *Arabidopsis* findings, low N activates BR signaling and induces the accumulation of de-phosphorylated BZR1 (active form) in *Solanum lycopersicum*, while BZR1-mediated BR signaling activates *ATGs* expression and promotes autophagosome formation, which act as a survival strategy during N deficiency and N starvation (Wang et al., 2019). Despite these advances, the downstream regulatory mechanism of crosstalk between BR signaling and NO_3_^-^ signaling remains to be deciphered.

In this study, we show that BES1 acts downstream BR signaling and Nitrate signaling and positively regulates lateral root elongation during nitrogen deficiency. Low concentration of nitrate can significantly up-regulate the transcript level of BES1, especially in root and promote de-phosphorylated BES1 (active) accumulation. Activated BES1 further promotes the root foraging response to low N through inducing the expression of key genes required for NO_3_^-^ uptake and assimilation. Moreover, we found that BES1 interacts with LBD37, a negative regulator of N availability signals (Rubin et al., 2009), and represses its binding to its converse binding site, and thus controls the nitrogen response. Our results reveal a novel mechanism by which BR signaling adjusts foraging response of root system to low N and they explain how BR signaling regulates nitrogen deficiency-induced root elongation.

## Materials and methods

### Plant materials and growth conditions

The basal medium was reported as previous study (Araya et al., 2014). KNO_3_ was added into the basal medium as the sole source of nitrogen at the indicated concentration in each experiment. The Columbia-0 (Col-0) background was used as wild-type Arabidopsis ecotypes in this study. *BES1* gain-of-function mutant *bes1-D* and *BES1* knock-down mutant *BES1-RNAi* were generously provided by professor Honghui Lin, and was originally generated by professor Yanhai Yin (Yin et al., 2002). 70% (v/v) ethanol and 0.1% (v/v) Triton X-100 was used to sterilize the Arabidopsis seeds. The sterilized seeds were dried in a laminar air flow and then plated on the basal medium supplemented with 10 mM KNO_3_. Solid medium plates containing seeds were vernalized at 4°C for 2 d in the dark, and then grown vertically under a long day photoperiod (Light/Dark: 16h/8h, 150 μmolm^-2^ s^-1^) at 22°C. Homogenous 5-day-old seedlings of each genotype were transferred to new basal medium plates as above mentioned but complemented with either 10 mM KNO_3_ (HN) or 0.5 mM KNO_3_ (LN). The seedlings were grown for another 9 days in either LN or HN medium for primary root length, lateral root length and number measurements, respectively. Statistical analysis was performed with one-way or two-way ANOVA analysis followed by Tukey’s test, P < 0.05.

### Plasmid constructs and transgenic plants

The coding regions of BES1 and LBD37 were amplified and cloned into the pCMBIA1300 (35S:X-GFP) and pCM1307-FLAG-HA (35S:FLAG-HA-X) vectors, respectively. These binary vectors were introduced into Agrobacterium tumefaciens strain GV3101 and then transformed into Col-0, *bes1-D*, *35S:BES1-GFP* and *BES1-RNAi* plants using the floral dip method as described previously (Zhang et al., 2006). For the interaction assays, coding regions of BES1and LBD37 were amplified and fused into pGADT7, pGBKT7, pMAL-c2X (N-MBP), pCAMBIA1300-NLuc (pNL), pCAMBIA1300-CLuc (pCL) vectors. For the luciferase assays, the ~2.0-kb promoters of *NRT2.1* was cloned and constructed into the pGREEN II-0800 (Promoter: LUC) vector. Oligo primers used for cloning are listed in **Supplementary Table S1**.

### Gene expression analysis

**Figure 1:**
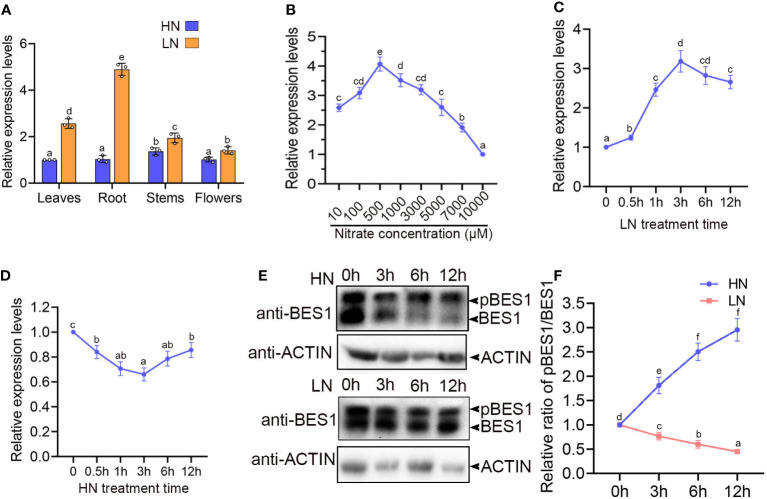
LN induces transcript level and dephosphorylation of BES1. **(A)** The relative expression of BES1 transcripts in the indicated tissues of wide-type plants (Col-0). 5-d-old Col-0 seedlings that grown on 1/2MS medium were transferred to medium with 0.5mM KNO_3_ (LN) or 10mM KNO_3_ (HN) for 5 weeks and then the different tissues (leaves, root, shoot and flower) were collected for gene expression analyses. The relative gene expression levels were presented as values relative to that of leaf sample. **(B)** Regulation of BES1 expression paten by nitrogen supply. Col-0 seedlings were grown on medium with various NO_3_^−^ concentrations (10, 100, 500, 1,000, 3,000, 5000, 7000 and 10000 μM) for 7 days and the *BES1* transcript levels in root tissues were analyzed by qRT-PCR. The relative gene expression levels were presented as values relative to that of the sample from 10000 μM NO_3_^−^. **(C, D)** Time courses of *BES1* expression in response to N availability. 7day-old Col-0 Seedlings grown on medium were treated by LN **(C)** or HN **(D)** for indicated times. The transcript levels of *BES1* were assessed in the roots by qRT-PCR. **(E)** Immunoblots showing the abundance of BES1 proteins in the root tissues of 1-week-old seedlings treated at HN (top panel) or LN (bottom panel) for the indicated time periods. BES1 was detected by western blot using anti-BES1 antibody. ACTIN2 was used as a loading control. pBES1 represents phosphorylated BES1. At least three times were repeated. **(F)** Quantitative results for Immunoblots results in **(E)**. The initial ratio of pBES1/BES1 of each treatment were set as ‘1’. In all gene expression analyses, *ACTIN2* was used as an internal standard. Data are presented as the mean ± SD (n = 3). In all results, different lowercase letters represent statistically significant differences (p < 0.05, two-way ANOVA).

For tissue expression paten analysis, the leaves and root were collected from the Col-0 plants grown on HN or LN conditions for 2 weeks while the stem and flowers were collected from Col-0 plants grown on HN or LN for 5 weeks. For nitrate concentration response analysis, wild-type plants were grown on nitrate concentration gradient medium (as described as in **Figure 1B**) for 14 days and then the root tissues were collected. For time course analysis, Col-0 plants were grown on medium containing 7mM KNO_3_ for 7 days and transferred to N-free medium for growing another 2 days, and then subjected to HN or LN treatment for 0.5h, 1h, 3h, 6h and 12h and finally the root tissues were collected. Total RNA was extracted from 0.1g of above materials using RNA extraction kit (Qiagen). The mRNA was reverse transcripted by cDNA using M-MLV Reverse Transcriptase kit (Invitrogen). The qRT-PCR was performed using TransStart Tip Green qPCR Super MixKit (Transgene, Beijing, China) and CFX Connect Real-Time System (Bio-Rad, Hercules, CA, USA). All data were collected from three replicates and normalized using ACTIN2 as the reference gene. All primer sequences used for qRT-PC were listed in **Supplementary Table S1**.

### ChIP qRT-PCR assay

ChIP was performed as described previously (Liang et al., 2020; Tan et al., 2021). Briefly, 4 g of *BES1-RNAi* and Col-0 seedlings or 2 g of tissue from Col-0 and *35S:BES1-GFP* seedlings was collected and cross-linked with 1% formaldehyde under vacuum. Add 0.125 mol/L glycine to stop the cross-linking. The samples with terminated cross-linking were rinsed three times using ddH_2_O and then ground to a fine powder in liquid nitrogen. The chromatin complex was isolated using Nuclear Extraction Buffer and sonicated at 4°C , and then incubated with 20 μg anti-GFP (Sigama) or BES1 antibody (Yanhai Yin Lab) overnight at 4°C . The immunoprecipitated protein-DNA complex were collected using 40 μL of protein beads (Invitrogen). The DNA fragments were isolated from protein-DNA complex *via* reversing cross-linking. The enrichment of DNA fragments was analyzed by qRT-PCR using the specific primers listed in **Supplementary Table S1**. All experiments were repeated at least three biological times.

### Yeast two-hybrid assays

Full-length cDNAs of LBD37/LBD38/LBD39 or BES1 were subcloned and fused into pGADT7 or pGBKT7 (Clontech) as prey or bait vectors. Both the bait and prey constructs were co-transformed into yeast AH109 cells according to the Yeast Transformation System 2 user manual (Clontech). The yeast clones were cultured on selective medium (SD/-Leu/-Trp/-His/-Ade, -LWHA) at 30°C for 3 days.

### Split-luciferase complementation assays

The split-luciferase complementation (split-LUC) assays were performed as described (Zhao and Zhou, 2020). Full length coding region of BES1 or LBD37 were PCR amplified and cloned into the pCAMBIA-1300-nLuc or pCAMBIA-1300-cLuc vectors, respectively. These vector constructs and control vectors were transformed into *Agrobacterium* strain GV3101. Overnight cultures of the bacterial that contained nLuc and cLuc constructs were mixed in equal ratios, and then the bacterial mixtures were infiltrated into the young leaves of *Nicotiana benthamiana* with infiltration medium (10mM MgCl_2_ 10mM MES, pH 5.7, 200 μM acetosyringone). After the infiltrated- *Nicotiana benthamianas* were grown in a long day condition (16h light/8dark) for 48h, their leaves were cut off and sprayed with 1 mM D-luciferin and then kept in dark for 5 minutes. The LUC signal was detected by a low-light cooled charge-coupled device camera (Tanon 5200, Tannon) with 15 minutes exposure time.

### Co-immunoprecipitation assays

Co-immunoprecipitation (co-IP) assays were performed as previously described procedures with modifications (Tan et al., 2018). Briefly, *35S:FLAG-HA*/Col-0 and *35S:FLAG-HA-LBD37*/Col-0 transgenic plants were grown on 1/2 MS medium for 14 days, and then the root tissues were harvested for co-IP assays. Total proteins were extracted from the root tissues with IP buffer containing 10mM Tris, pH 7.5, 0.5% Nonidet P-40, 2 mM EDTA, 150mM NaCl, 1 mM PMSF, and 1% plant protease inhibitor cocktail (Amresco). The extracts were centrifuged at 16000rpm for 15 min, the supernatants were incubated with HA agarose beads (Sigma-Aldrich) in IP buffer at 4°C for 2h. The beads were then collected and washed for five times with IP buffer. The beads were collected and boiled in 2x SDS loading buffer. The samples were separated in 10% SDS-PAGE and analyzed by immunoblotting sing anti-HA antibody, and the coimmunoprecipitated proteins were detected with anti-BES1 antibody. Samples immunoprecipitated from the *35S:FLAG-HA*/Col-0 plants were used as a negative control.

### Electrophoresis mobility shift assays

EMSAs were performed as previously described procedures with modifications (Tan et al., 2018; Tan et al., 2021). Briefly, MBP-LBD39 and HIS-BES1 fusion proteins were expressed in *E. coli* host strain BL21 (DE3) and purified for use in EMSA. The DNA probes (**Supplementary Table S1**) containing the putative binding site or mutant binding site were synthesized and then incubated together with MBP-LBD37 or MBP-LBD37 + HIS-BES1 in 20 μL reaction system containing 5mM HEPES KOH, pH 8.0, 50mM KCl, 1 mM dithiothreitol (DTT) and 10% glycerol on ice for 60 min. Then, the samples were separated on 5% native polyacrylamide gels in 0.53 TBE buffer. The detection of protein-DNA interactions was performed as the Light Shift Chemiluminescent EMSA Kit (cat. no. 20148; Thermo Fisher) manual.

### Transient transcription assay

Transient transcription Luc assays were performed as previously described with modification (Tan et al., 2021). *35S:FLAG-HA-BES1* or *35S:FLAG-HA-LBD37* was used as a effector and the pGreen II 0800-LUC vector (Hellens et al., 2005) harboring *pNRT2.1* and *35S:REN* was used as a reporter construct. Isolated protoplasts from Col-0 leaves were transfected with 5 μg reporter construct and 10 μg of each effector construct and cultured in dark for 16h. The luciferase activity was measured by dual luciferase assay reagents (Promega, Madison, WI, USA) using a luminometer (erthold Centro LB960). Relative luciferase activity was normalized to REN activity and the relative LUC/REN ratio was used as the final measurement.

### Quantification and statistical analysis

Statistical analyses were performed using GraphPad PRISM and SPSS software. ANOVAs were performed to check for significant differences in multiple comparisons in Figures. Different letters represent statistical significantly analyzed by ANOVA (P<0.05) for multiple comparisons and the same letters represent the levels that have no significant difference.

## Results

### Transcript level and dephosphorylation form of BES1 are accumulated under low N condition

Given the close relationship between BR signaling and Nitrogen foraging response (Jia et al., 2019; Jia et al., 2020), we examined the master regulator of BR signaling pathway, BES1, also acts as an important downstream regulator in low N response. Firstly, we conducted a gene-expression survey of *BES1* in response to high concentration N (HN, 10mM) and mild low concentration N (LN, 0.5 mM NO_3_^-^) in different tissues of Arabidopsis. The results showed that *BES1* mRNA is accumulated in all tissues of *Arabidopsis*, especially higher in root under low N condition, compared with that under HN condition (**Figure 1A**). We further detected the transcriptional levels of *BES1* in roots of the seedlings grown with various concentration of NO_3_^-^ supplies for 14 days. In this experiment, we found that mild N deficiency (LN) can significantly induce *BES1* expression, but high N can dramatically suppress BES1 accumulation (**Figure 1B**). We also analyzed the expression levels of *BES1* upon HN or LN treatment at different times. The transcript level of *BES1* significantly accumulated as early as 0.5 h and reached at a peak after 3h in the roots of Col-0 exposed to LN condition (**Figure 1C**). When exposed to HN conditions, BES1 expression decreased rapidly and reached at a lowest value after 3h (**Figure 1D**). The phosphorylation status of BES1 is a central indicator of BR signaling (Yin et al., 2002; Nolan et al., 2020). We then examined the phosphorylation status of BES1 protein using BES1 antibody by immunoblot analysis in WT plants under different N supplies. When grown in HN condition, the ratio of phosphorylated BES1 and dephosphorylated BES1 (pBES1/BES1) were significantly increased, while it is dramatically decreased after LN treatment, suggesting that HN promotes BES1 phosphorylation, but LN induces the accumulation of dephosphorylated BES1 (**Figures 1E, F**). These results indicated that both transcriptional level and activity of BES1 were promoted by mild N deficiency.

### BES1 positively regulates lateral root elongation in response to low N

**Figure 2:**
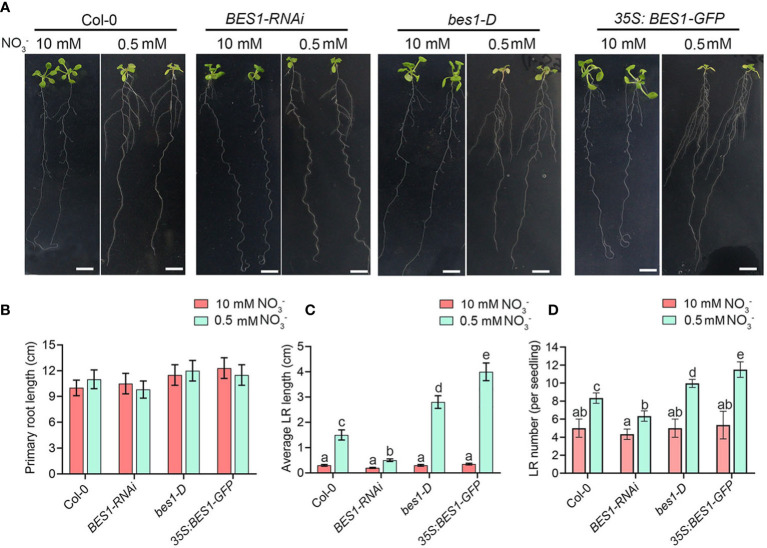
BES1 promotes the lateral root growth and development at LN. **(A)** Seedling phenotypes of Col-0, *BES1-RNAi*, *bes1-D* and *35S:BES1-GFP*. 5-day-old seedlings were precultured on medium containing 10 mM NO_3_^-^ and then transferred to HN medium or LN medium for another 9 days for quantification of primary root length **(B)**, average lateral root length **(C)**, and lateral root number **(D)**. Error bars indicate the s.d. (n = 10-15). Different Lowercase letters above the bars indicate statistically significant differences between samples (p < 0.05, two-way ANOVA). Scale bars, 1cm.

Because BES1 is a nitrate-responsive regulator, we further explored the function of BES1 in regulation of LN responses, especially the root forging responses under LN condition, the root system characters were analyzed in Col-0, *BES1* RNA interference (*BES1-RNAi*), gain-of-function mutant *bes1-D* and overexpression plant (*35S:BES1-GFP*). Under HN condition, we detected no differences in the primary root, lateral root length and lateral root number between WT and *BES1* mutants (**Figures 2A–D**). At LN, the primary root length was no significant difference in all genotype plants, while both the lateral root growth and development were significantly increased compared with that at HN (**Figures 2A–D**). However, the lateral root elongation and lateral root number in *BES1-RNAi* plants were only slightly increased, while *bes1-D* and *35S:BES1-GFP* exhibit strong stimulatory responses, compared with WT (**Figures 2A–D**). These data showed that BES1 is involved in the mild N deficiency-induced lateral root responses and enhanced the foraging response of plants to low N.

### BES1 suppresses the expression of repressors in nitrate signaling pathway and promotes the expression of genes involved in nitrate response

Given the rapid induction of BES1 by mild N deficiency and the vital roles of BES1 in lateral root elongation in response to low N, we speculated that BES1 is participated in regulation of nitrate signaling-responsive genes expression. According to previous ChIP-chip sequence studies and BES1-regulated genes transcriptome (Sun et al., 2010; Yu et al., 2011), we found LBD37 and its two homologous LBD38/LBD39, which were reported to act as repressors in nitrate signaling pathway (Rubin et al., 2009), were direct targets of BES1 and BZR1. We then detected the transcript accumulation of these repressors in *BES1*-related mutants.

**Figure 3:**
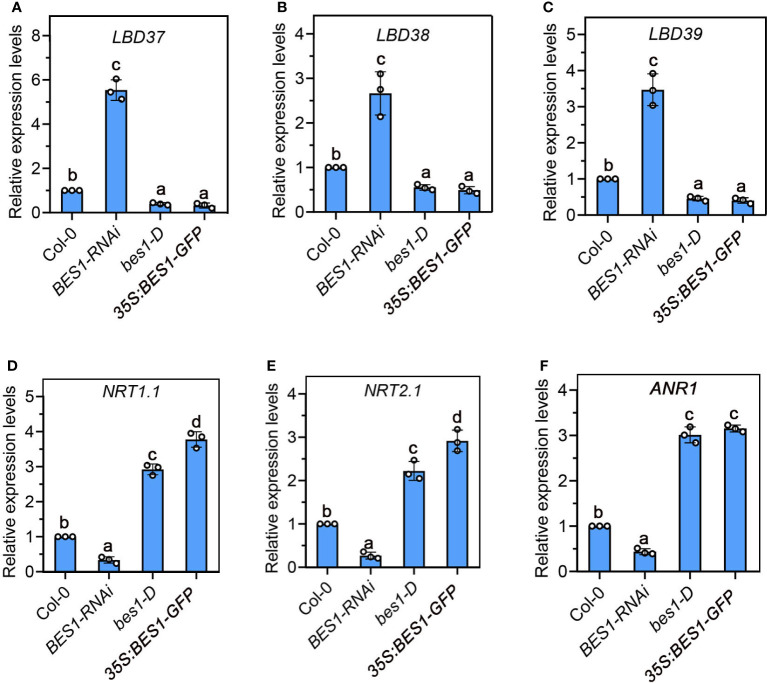
Expression of nitrate signaling repressors, *LBD37/38/39* **(A-C)**, and LN-responsive genes *NRT1.1* **(D)**, *NRT2.1* **(E)** and *ANR1* **(F)** in the roots of 7-day-old Col-0, *BES1-RNAi*, *bes1-D* and *35S:BES1-GFP* at LN. The expression levels of genes in the Col-0 seedlings were set to 1.00. Error bars represent s.d. of three biological repeats. Different Lowercase letters above the bars indicate statistically significant differences between samples (p < 0.05, one-way ANOVA).

Our gene expression analysis showed that the expression of *LBD37/LBD38/LBD39* were significantly upregulated in *BES1-RNAi* roots and significantly suppressed in *bes1-D* and *35S:BES1-GFP* roots under low N condition in comparison to WT (**Figures 3A-C**). LBD37/LBD38/LBD39 can suppress a class of known N-responsive genes, including several key genes involved in NO_3_^-^ uptake and assimilation, such as *NRTs*, *ANR1*, and *NIAs*. We then detected the expression of downstream targes of LBD genes that were reported to be involved in root system response to LN, including *NRT1.1*, *NRT2.1* and *ANR1* (Rubin et al., 2009). The results showed that the expression of all these three genes were repressed in *BES1-RNAi* roots, while their expressions were strongly upregulated by enhancement of BES1 function (**Figures 3D–F**). These results provide evidence that BES1 negatively regulates nitrate signaling repressors and positively modulates nitrate-responsive genes involved in nitrate transport and lateral root foraging responses.

### BES1 binds to the promoters of LBD37/38/39 in a BR- and LN-enhanced manner

**Figure 4:**
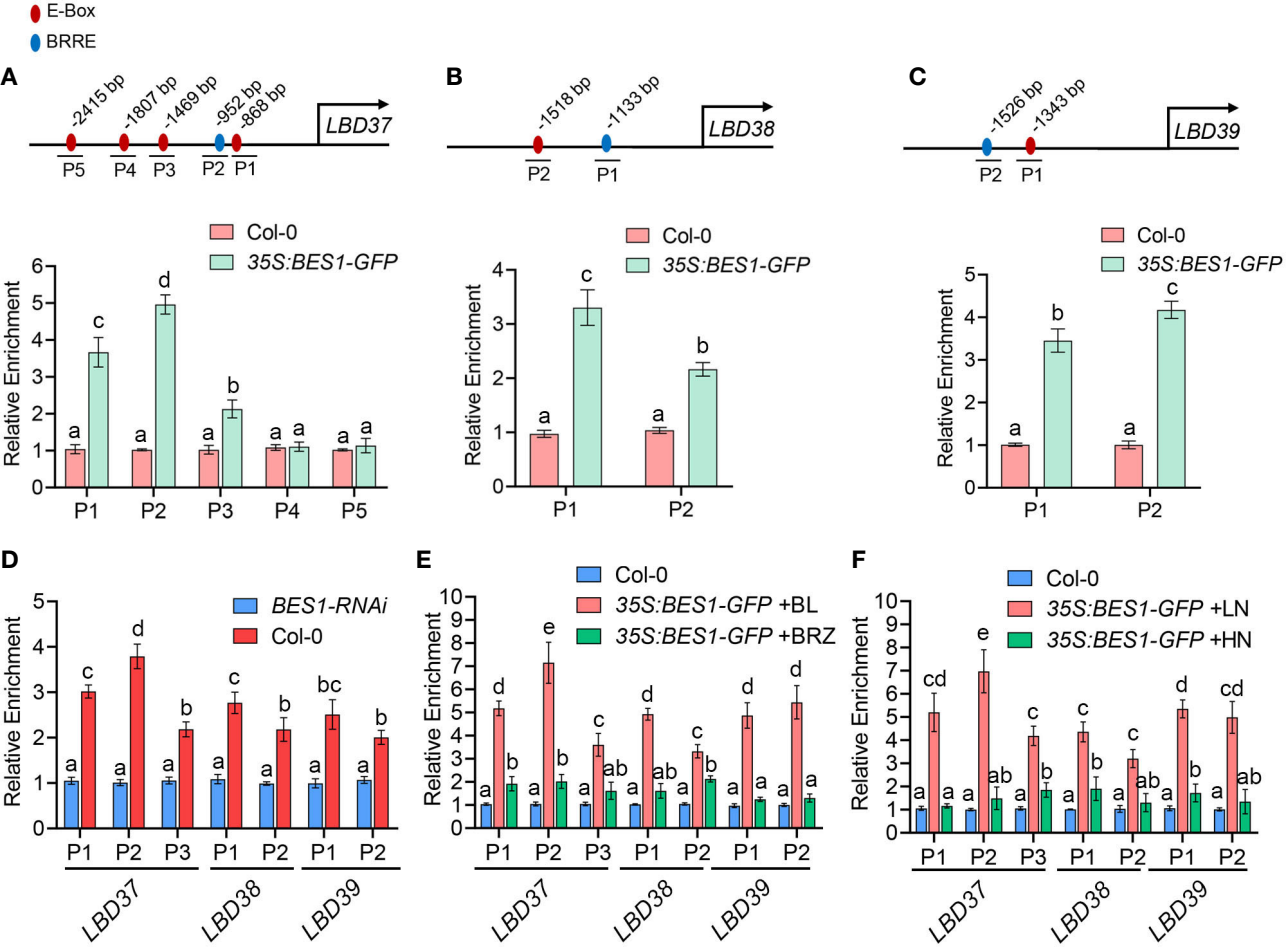
ChIP-qPCR showing BES1 binds to BRRE and E-box of *LBD37/38/39* promoters in a BR- and LN-enhanced manner. **(A-C)** Promoter analysis of LBD37/38/39 promoters (top panels) and ChIP-qPCR (bottom panels). The E-box elements were showed as red circles and BRRE elements were showed as blue circles in the LBD37/38/39 promoters. P1-P5 represent the perfect E-box or BRRE sites. The numbers indicate relative position of the E-box or BRRE elements in the respective promoters relative to the ATG start codon. The ChIP assays were performed with chromatin prepared from 14-d-old Col-0 and *35S:BES1-GFP* transgenic plants. The enrichment of DNA was calculated as the ratio between IP and Input, and TA3 was used as internal control. The enrichment level of Col-0 was set to 1.00. **(D)** The ChIP-qPCR assays were performed with chromatin prepared from Col-0 and *BES1-RNAi* plants using an anti-BES1 antibody-coupled Agarose beads (IP). The enrichment of DNA was calculated as the ratio between IP and Input, and TA3 was used as internal control. The enrichment level of *BES1-RNAi* was set to 1.00. **(E)** BR promotes the binding affinity of BES1 to LBDs promoters. 12-day-old Col-0 and *35S:BES1-GFP* seedlings with BL or BRZ treatment for 2 days were used to ChIP-qPCR assays using anti-GFP-Agarose beads. The enrichment of DNA was calculated as the ratio between IP and Input, and TA3 was used as internal control. The enrichment level of Col-0 was set to 1.00. **(F)** LN enhances the binding of BES1 to the promoters of LBDs. 7-old-day Col-0 and *35S:BES1-GFP* seedlings grown on normal medium were transferred to HN or LN medium for another 7 days and then the roots of these plants were collected for ChIP-qPCR assays using anti-GFP agarose. The enrichment of DNA was calculated as the ratio between IP and Input, and TA3 was used as internal control. The enrichment level of *BES1-RNAi* was set to 1.00. In the above results, error bars represent standard deviations of three biological repeats and Different Lowercase letters above the bars indicate statistically significant differences between samples (p < 0.05, two-way ANOVA).

Since LBD37/38/39 were targets of BES1 in previous ChIP-chip data (Sun et al., 2010; Yu et al., 2011) and their expression were repressed by BES1, we then investigated whether BES1 control these genes expression by directly binding to their promoters. Several earlier studies showed that BES1 can bind to BRRE elements (CGTG^T/C^G) and E-box (CANNTG) to control BR target genes (Sun et al., 2010; Yu et al., 2011). Therefore, we analyzed the promoters of LBD37/38/39 genes (3kb upstream of the initiation codon) and found several E-box and BRRE elements (**Figures 4A–C**). We then examined the binding of BES1 to promoters of LBD37/38/39 by chromatin immunoprecipitation (ChIP) assays using *35S:BES1-GFP* transgenic plants. We first used GFP antibody to immunoprecipitate the protein-DNA complexes and then detected the enriched DNA sequence with the primer pairs covering *LBDs* promoter regions. As shown in **Figure 4A–C**, BES1 can bind to part of the E-box and BRRE motif of these LBD promoters. We then conducted a ChIP experiment using BES1 antibody to confirm the binding ability of native BES1 to *LBD37/38/39* promoters. As shown in **Figures 4D**, the native BES1 indeed could bind to the promoters of LBD37/38/39, which is consistence with the results of ChIP assays using GFP antibody (**Figure 4D**).

In the classical BR signaling pathway, BR can induce the dephosphorylation of BES1 and dephosphorylated BES1 bind to BR-responsive genes promoters and control their expression (Yin et al., 2002). Thus, we further investigated whether BR promotes BES1 binding to LBDs promoter. We grow the *35S:BES1-GFP* plants in presence of BL (the most active form of BRs) to promote BES1 dephosphorylation or in the presence of BRZ (the special inhibitor of BRs biosynthesis) to induce the phosphorylation of BES1 and then conducted ChIP-qPCR assays (**Supplementary Figures S1A, B**). Compared to the *35S:BES1-GFP* plants treated with BRZ, dephosphorylated BES1 in BL-treated plants has a higher binding affinity for LBDs promoters (**Figure 4E**; **Supplementary Figures S1A, B**). As our above experiments showed LN can induce dephosphorylation of BES1 and HN can induce BES1 phosphorylation, we further investigated the effects of LN or HN on the binding ability of BES1 to the LBDs promoters (**Supplementary Figures S1C, D**). Consistent with the results of BL treatment, LN also can promote the binding ability of BES1 to LBDs promoters, while HN significantly repressed BES1 binding to LBDs promoters like the situation of BRZ treatment (**Figure 4F**). Taken together, these results suggest that BES1 directly controls LBD genes in a BR-and LN- enhanced manner.

### BES1 interacts with LBD37/LBD38/LBD39 transcription factor

**Figure 5:**
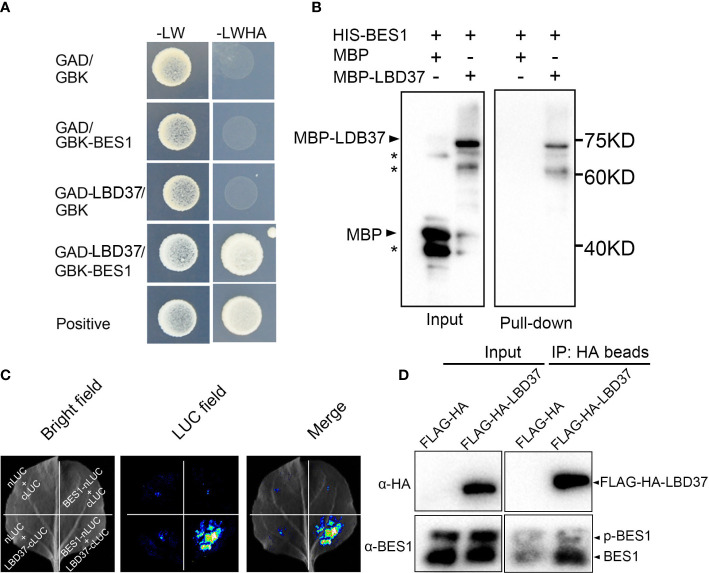
BES1 interacts with LBD37. **(A)** Yeast two hybrid assays. The pGADT7- (shown as GAD) and pGBKT7- (shown as GBK) derivative constructs were shown on the left side. The interaction was selected on the Leu, Trp, His, and Ade dropout medium (-LWHA). **(B)** HIS pull-down assay. *represents non specific band. BES1 was fused with HIS tag, LBD37 was fused with MBP tag. After coincubation of HIS-BES1 and MBP-LBD37, the proteins were immunoprecipitated with HIS agarose beads. The pull-down protein was detected with anti-MBP antibody. MBP was used a negative control. **(C)** Split-Luc assays showing BES1 interacting with LBD37 *in vivo*. BES1 and LBD37 were fused with nLUC and cLUC of luciferase, respectively. Empty vectors were used as negative control. **(D)** Co-immunoprecipitation (Co-IP) assays. Total proteins from *35S:FLAG-HA* or *35S:FALAG-HA-LBD37* plants were immunoprecipitated using anti-HA beads and detected by immunoblotting with anti GFP or anti BES1 antibody, respectively. The input of proteins for immunoprecipitation was shown on left panel.

As BES1 always directly regulates and interacts with multiple cofactors to modulated BR target genes, we then test if BES1 can interact with its targets LBD37/38/39. We employed yeast two-hybrid to examine the interaction, the coding region of LBD37 was introduced into the GAL4 activation domain of the prey vector (GAD-LBD37) and the full length of BES1 was fused with the GAL4 binding domain of bait vector (GBK-BES1). Yeast two-hybrid assays showed that BES1 can interact with LBD37 in yeast (**Figure 5A**). We also detected the interaction of BES1 and other two homologous of LBD37, LBD38/39, by Y2H assays. As the results shown, BES1 indeed interact with both LBD38 and 39 (**Supplementary Figure S2**). As the functional redundancy of LBD37, LBD38 and LBD39 in nitrate signaling pathway, we selected LBD37 to conduct further investigation. We then performed an *in-vitro* pull-down assay to confirm the interaction of BES1 with LBD37 and found that HIS-BES1 could pulldown MBP-LBD37 protein (**Figure 5B**). We further tested the physical interaction of BES1 with LBD37 using split-luciferase complementation (split-LUC) assays, the results showed that BES1 interacts with LBD37 *in vivo* (**Figure 5C**). Finally, the co-immunoprecipitation assay was performed using *35S:FLAG-HA-LBD37* transgenic plants. The results showed that FLAG-HA-LBD37 immunoprecipitated with anti-HA antibodies can almost only pulldown dephosphorylated form of BES1, indicating that LBD37 mainly interacts with dephosphorylated BES1 (active) *in vivo* (**Figure 5D**). Taken together, these results suggest that BES1 can interact LBD37.

### BES1 inhibits the DNA-binding Activity of LBD37

**Figure 6:**
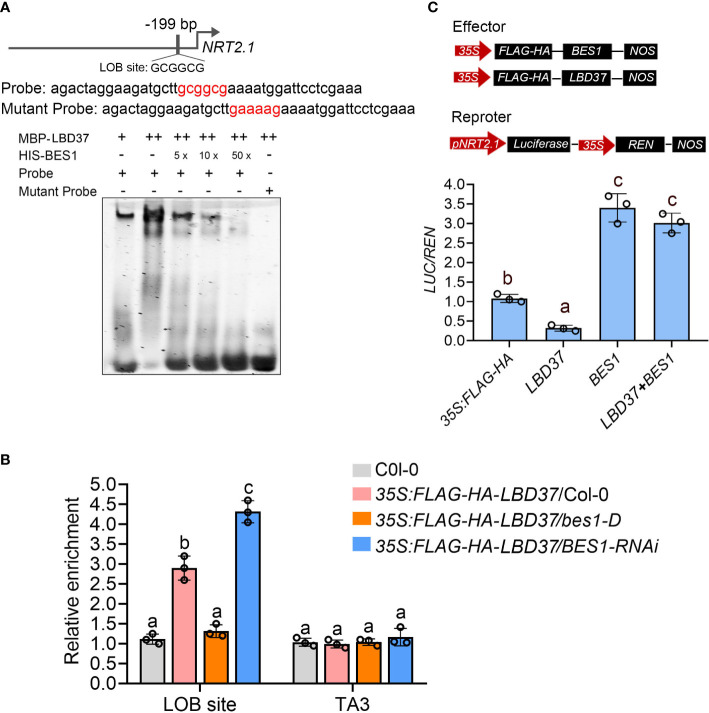
BES1 inhibits LBD37 binding to its target gene. **(A)** EMSA showing BES1 inhibits the DNA binding ability of LBD37. The top panel showed the DNA sequences used as DNA probe containing the normal or mutated form of LOB site (GCGGCG). The bottom panel showing LBD37 bind to *NRT2.1* promoter and addition of BES1 with LBD37 suppresses the DNA binding ability of LBD37. **(B)** ChIP-qPCR assay showing BES1 inhibits LBD37 binding to LOB site *in vivo*. The wild-type (Col-0), *35S:FLAG-HA-LBD37/Col-0*, *35S:FLAG-HA-LBD37/bes1-D* and *35S:FLAG-HA-LBD37/BES1-RNAi* were used to prepare chromatin and ChIP assay with anti-HA Agarose beads. The ChIP products were used to detect promoters containing LOB site. error bars represent standard deviations of three biological repeats and different Lowercase letters above the bars indicate statistically significant differences between samples (p < 0.05, two-way ANOVA). **(C)** The transient transcriptional assay showing BES1 inhibits the binding activity of LBD37 on *NRT2.1* promoter. The top panel showed the structures of *pNRT2.1:Luc* and two effector constructs. BES1, LBD37 and REN are driven by 35S promoter. The bottom panel showed the *LUC/REN* ratio calculated by *pNRT2.1:Luc* relative to the REN activity. Error bars represent SDs of three biological repeats. Different Lowercase letters above the bars indicate statistically significant differences (p < 0.05) determined by one-way ANOVA.

LBD37 is a member of plant-specific ASYMMETRIC LEAVES2 (AS2)-LIKE (ASL)/LATERAL ORGAN BOUNDARY (LOB) DOMAIN (LBD) gene family, which was previously reported to recognize a consensus LOB motif (GCGGCG) by forming a regulatory complex with bHLH proteins (Husbands et al., 2007). Several genes functioning in nitrate uptake and assimilation, including *NRT1.1*, *NRT2.1*, *ANR1*, *NIA1* and *NIA2*, were regulated by LBD37 and its two close homologs LBD38 and LBD39 (Rubin et al., 2009). To further explore how BES1 and LBD37 coregulate these N-responsive genes, we analyzed the promoters of *NRT1.1*, *NRT2.1* and *ANR1* which are upregulated by BES1(**Figure 3A**) but repressed by LBD37 (Rubin et al., 2009). There is only one LOB motif in upstream 199bp of *NRT2.1*’ promoter whereas no LBD binding site on the promoters of *NRT1.1* and *ANR1*, so we selected *NRT2.1* to further investigation (**Figure 6A**). Electrophoretic mobility shift assay (EMSA) was performed using recombinant BES1 and LBD37 proteins along with a *NRT2.1* promoter fragment containing LOB motif element as DNA probe (**Figure 6A**). The results showed that LBD37 bound to the promoter region of *NRT2.1* but could not bind to the mutated LOD motif (**Figure 6A**). When BES1 was present, the binding affinity of LBD37 to the *NRT2.1* promoter was dramatically repressed (**Figure 6A**). Next, we performed ChIP-qPCR to examine whether LBD37 bound to the *NRT2.1* promoter *in vivo* and whether BES1 affected the DNA-binding ability of LBD37. LBD37 specifically bound to the *NRT2.1* promoter, while this binding was abolished in *bes1-D* and significantly enhanced in *BES1-RNAi* plants (**Figure 6B**). Finally, Luciferase transient transcription assay was conducted to confirm the effect of BES1 on LBD37 transcriptional activity. Different combinations of effectors were coexpressed, followed by sequential recording of luminescence from firefly luciferase driven by the 2-kb *NRT2.1* promoter (*pNRT2.1:LUC*). The results showed that LBD37 alone significantly repressed the expression of *NRT2.1*, while BES1 alone had a promoting effect (**Figure 6C**). When coexpressing LDB37 and BES1 with reporter, BES1can significantly inhibit the activity of LBD37 on the promoter of *NRT2.1* (**Figure 6C**). Taken together, these results suggest that BES1 interacts with LBD37 and suppresses its binding activity on target gene.

### BES1 suppresses the inhibitory effect of LBD37 on lateral root growth in LN

**Figure 7:**
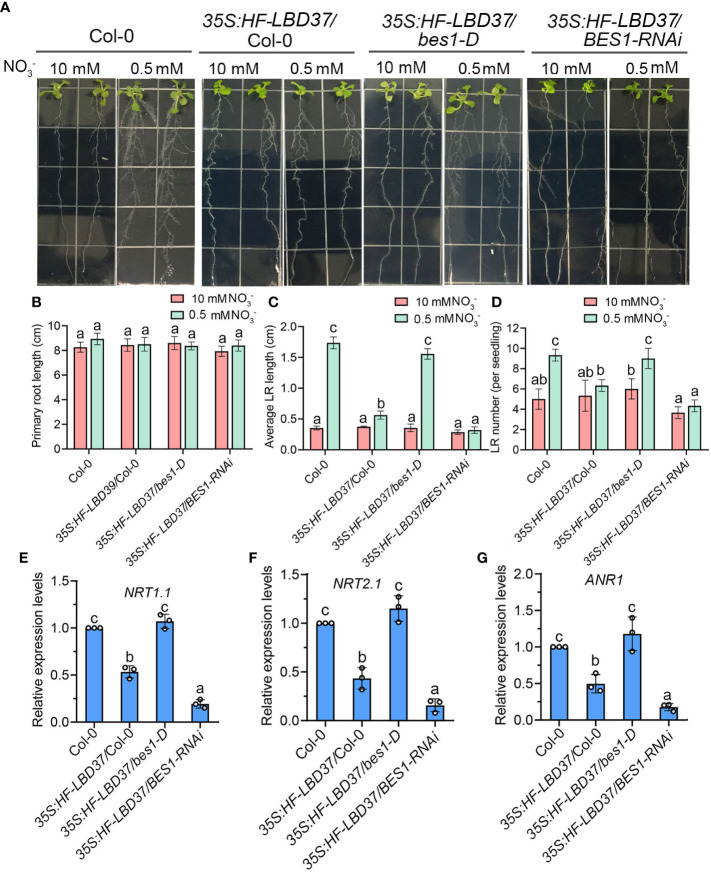
BES1 suppresses the inhibitory effect of LBD37 on lateral root growth in LN. **(A)** Seedling phenotypes of *35S:FLAG-HA-LBD37/*Col-0, *35S:FLAG-HA-LBD37/bes1-D* and *35S:FLAG-HA-LBD37/BES1-RNAi* plants. 5-day-old seedlings were precultured on medium containing 10 mM NO_3_^-^ and then transferred to HN medium or LN medium for another 9 days for quantification of primary root length **(B)**, average lateral root (LR) length **(C)**, and lateral root (LR) number **(D)**. Error bars indicate the s.d. (n=10-15). Different Lowercase letters above the bars indicate statistically significant differences between samples (p < 0.05, two-way ANOVA). **(E-F)** Expression analysis of LN-responsive genes *NRT1.1* **(E)**, *NRT2.1* **(F)** and *ANR1* **(G)** in the roots of 7-day-old Col-0, *35S:FLAG-HA-LBD37/*Col-0, *35S:FLAG-HA-LBD37/bes1-D* and *35S:FLAG-HA-LBD37/BES1-RNAi* plants at LN. The expression levels of genes in the Col-0 seedlings were set to 1.00. Error bars represent s.d. of three biological repeats. Different Lowercase letters above the bars indicate statistically significant differences between samples (p < 0.05, one-way ANOVA).

To further prove that BES1-LBD37 module is involved in LN-mediated root response, we overexpressed LBD37 in Col-0, *bes1-D*, and *BES1-RNAi* respectively. Overexpression of LBD37 in Col-0 results in a severe inhibitory effect on both LN-induced lateral root elongation and lateral root number increase (**Figures 7A–D**). However, the LN-induced lateral growth and development was significantly enhanced in *35S:FLAG-HA-LBD37/bes1-D* and compared to overexpressing LBD37 in Col-0, while the phenotype of *35S:FLAG-HA-LBD37/BES1-RNAi* was similar to that of *BES1-RNAi* and *35S:FLAG-HA-LBD37*/Col-0 (**Figures 7A–D**). We also detected the accumulation of LBD37 in *35S:FLAG-HA-LBD37*/Col-0, *35S:FLAG-HA-LBD37/bes1-D* and *35S:FLAG-HA-LBD37/BES1-RNAi*, and found that both the transcript and protein level of LBD37 in these all transgenic plants were similar (**Supplementary Figures S3A, B**). We also constructed a *35S:FLAG-HA-LBD37/35S:BES1-GFP* transgenic plants to confirm the effect of BES1 on LBD37-repressed lateral root growth in response to LN, the similar LN-responsive phenotypes of lateral root was observed between *35S:FLAG-HA-LBD37/bes1-D* and *35S:FLAG-HA-LBD37/35S:BES1-GFP* (**Supplementary Figures S4A–D**). These results suggest that LBD37 negatively regulates lateral root growth in response to LN and BES1 can inhibits the function of LBD37 in lateral root foraging response.

To determine whether BES1 and LBD37 antagonistically regulate nitrate-regulated genes expression, we performed qRT-PCR in Col-0, *35S:FLAG-HA-LBD37*/Col-0, *35S:FLAG-HA-LBD37/BES1-RNAi,35S:FLAG-HA-LBD37/bes1-D* and *35S:FLAG-HA-LBD37/35S:BES1-GFP* under LN condition. As shown in **Figures 7E–G**, the expressions of nitrate transporter genes *NRT1.1*, *NRT2.1* and nitrate signaling gene *ANR1* were downregulated by LBD37. These genes transcript levels were much higher in *35S:FLAG-HA-LBD37/bes1-D* and *35S:FLAG-HA-LBD37/35S:BES1-GFP* compared with *35S: FLAG-HA-LBD37*/Col-0, while further repressed by loss-function of BES1 (**Figures 7E-G**; **Supplementary Figures S4E–F**). Taken together, these results indicate that BES1 and LBD37 differently control nitrate-regulated genes expression.

## Discussion

**Figure 8:**
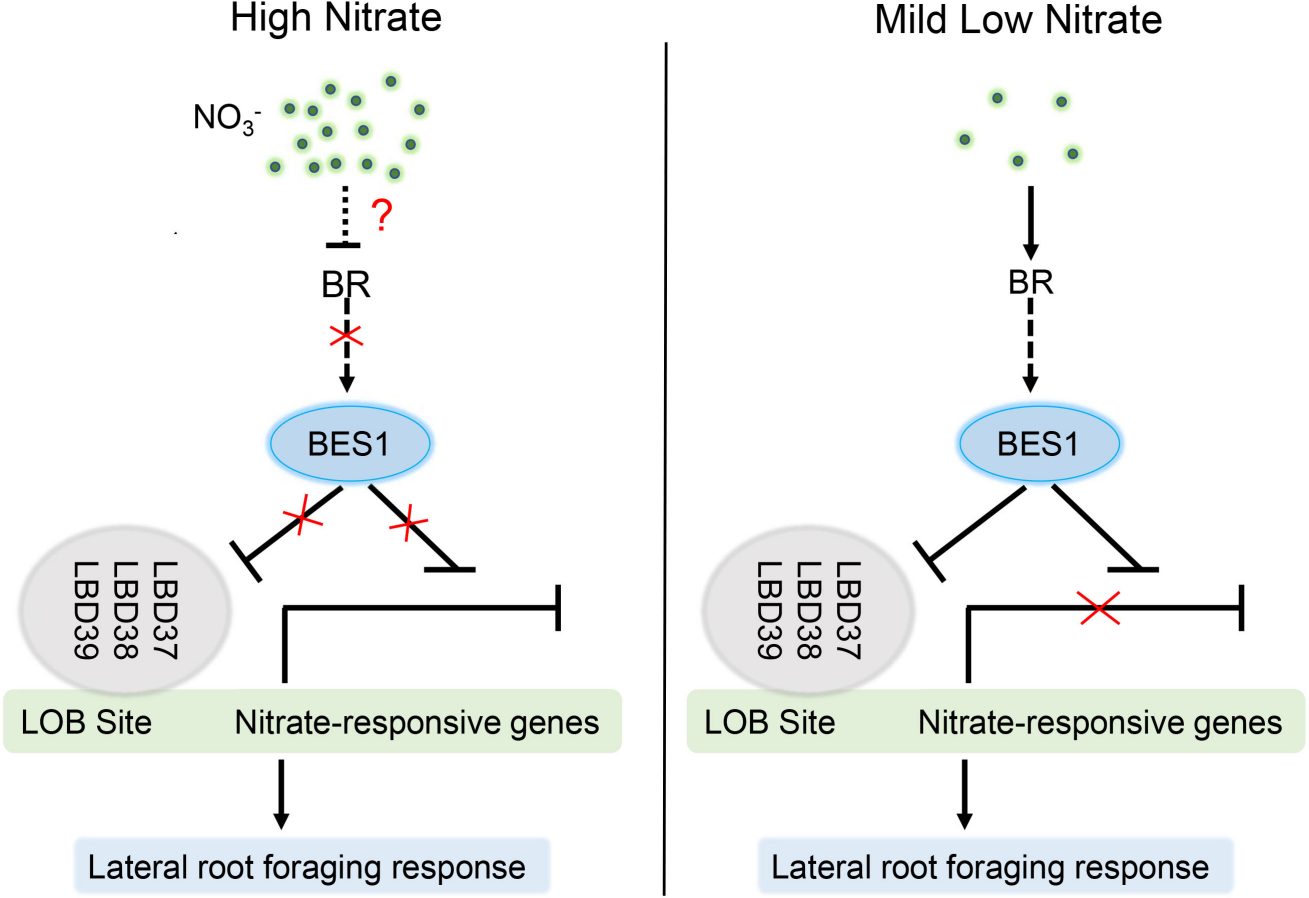
Proposed model to illustrate how BES1-LBDs cascade integrates BR and LN response to precisely fine-tune lateral root growth. Under HN conditions, high concentration of nitrate inhibits BR level and promotes the phosphorylation of BES1 (inactivation) through an unknown mechanism. Inactivated BES1 failed to interact with LBD37/38/39 or suppresses their expression, resulting in binding of LDBs to LN-responsive genes promoters and reduction of their transcript level and thus inhibitory of lateral root growth. At mild nitrogen deficiency, LN induces the accumulation of BES1 transcript and dephosphorylation *via* promoting BR biosynthesis. Activated BES1 can suppress the expression and DNA binding ability of LBDs, and thus enhance the expression of LN-responsive genes, which stimulate lateral root growth and development in response to LN, commonly described as nitrogen foraging response.

Nitrogen and nitrate (the primary nitrogen source in natural) are essential macronutrients for plants and act as an important molecular signal that regulates a range of genes expression to control plant growth, development, physiology and metabolism. Especially, the response of Root System Architecture (RSA) is crucial for plant optimizing nitrogen capture in the natural environments with disparate nutrient availability. RSA is profoundly affected by a complex regulatory network of hormonal and nitrate signaling pathways. Several studies have shown that nitrate acts as both local signal and systemic signal to regulate biosynthesis, degradation, transport, and signaling of different phytohormones to adjust root system modification (Vidal et al., 2010; Ristova et al., 2016; Vega et al., 2019; Hu et al., 2021). Recently, connections between nitrate responses and Brassinosteriod have been proposed by Genome Wide Association Studies using the Arabidopsis thaliana accessions. The substitution of 319 leucine with proline (319L to P) in BSK3 kinase domain can enhance BR sensitivity and signaling to promote plant root elongation under mild low nitrogen conditions. In addition, low N specifically promotes the expression of the BR co-receptor BAK1 and stimulate RSA growth by BSK3-BAK1 module (Jia et al., 2019). Furthermore, mild N deficiency can also induce key genes in BR biosynthesis and natural variation in BR synthesis contributes to the root foraging response (Jia et al., 2020). Despite these progresses in the studies of the crosstalk between BRs and nitrogen responses, the downstream regulatory mechanisms by which connecting BR signaling and nitrogen responses are not well understanding. Here, we show that mild nitrogen deficiency induces and activates BES1, the central regulator in BR signaling pathway, through activating BR biosynthesis and signaling and that the activated BES1 can induces the transcript levels of nitrate-responsive genes through inhibiting both the expression and DNA binding activity of LBD37 and thus contributes to the root foraging response at low N condition. However, when grown in HN conditions, high concentration nitrate may repress BR though an unknown mechanism, and thus led to phosphorylation of BES1, resulting in inactivation and degradation of BES1, and inhibitory of the lateral root foraging response (**Figure 8**).

BRs are a class of steroid hormones involved in regulation of plant growth, development and adaptions of plants to environmental conditions, which are commonly considered as a growth-promoting phytohormones, as its promotion roles in hypocotyl elongation and the dwarf phenotypes of BR-deficient and BR-insensitive mutants (Guo et al., 2013; Nolan et al., 2020). Studies revealed that BRs acts fundamental roles in regulating of root growth and development in response to the fluctuating environmental cues, such as gravitropic response meristem size maintenance, lateral root initiation, root hair formation, and cell wall orientation in root meristem (KIM et al., 2007; Hacham et al., 2011; Gupta et al., 2015; Li et al., 2020; Li et al., 2021). However, BRs regulate root growth in response to multiple environmental cues through complicated manners. High salinity can inhibit BZR1 nuclear accumulation and BR signaling functions, which leads to growth quiescence in roots (Geng et al., 2013), while elevated ambient temperature leads to increased root elongation by inhibiting BR signaling (Martins et al., 2017). Additionally, BR was reported to modulate plant nutrient availability in the soil microenvironment. Iron deficiency activates BR signaling to promote root growth while the iron distribution in Arabidopsis roots was similarly affected by perturbed BR signaling. In contrast, lack of phosphate cause BR signaling activation and subsequent root growth *via* enhancing iron accumulation (Singh et al., 2018). A recent study found low boron repressed the BR biosynthesis genes and the active BR accumulation, which leads to the inhibitory of primary root growth. BR signaling gain-of-function mutants *bes1-1D/bzr1-1D* seedlings exhibited insensitivity to boron deficiency and longer primary root length under similar conditions (Zhang et al., 2021). Our results suggest that the promotion roles of BR signaling in lateral root growth is dependent on the accumulation and activity of BES1, as gain-of-function BES1 mutant *bes1-D* can significantly enhance the LN-induced lateral elongation, while knock down of BES1 results in insensitivity of lateral root to LN (**Figure 2**). Thus, BR signaling affects many aspects of root growth and development in response to various environmental cues by the different components.

As a central regulator of BR signaling pathway, BES1 plays an important role in integrating BR signaling with other pathways (Kono and Yin, 2020; Nolan et al., 2020). For example, BES1 modulates the crosstalk of BR signaling and light signaling, drought response and thermotolerance (Oh et al., 2012; Ye et al., 2017; Albertos et al., 2022). Several lines of evidence from our study suggest that BES1 links BR signaling and nitrate signaling pathway to regulate root system. Firstly, LN treatment induces and HN slightly represses BES1 expression in root. Further, LN can also promote dephosphorylated BES1 accumulation, while HN inhibit the dephosphorylation of BES1. Second, BES1 is involved in regulation of low nitrate-stimulated lateral root growth and development. The mutant lacking BES1 displayed a similar root phenotype in root system at HN condition but exhibited deficiency in LN-stimulated lateral root elongation and development. Gain-of-function or overexpression of BES1 led to increase lateral root growth and development at LN. However, we have not observed the phenotype of primary root growth at both HN and LN in all genotype plants, which may be due to nitrate used as the sole nitrogen source. Third, BES1 can directly regulate several nitrate response-related genes expression. LBD37/38/39, three repressors of nitrate signaling are identified as BES1/BZR1 target transcriptional factors in previous ChiP-Seq data (Sun et al., 2010; Yu et al., 2011). In our results, these repressors expression was downregulated in BES1 gain-of-function mutant or overexpression plant, while it was upregulated by BES1 knock-down. In addition, the key genes involved in nitrate foraging responses including *NRT1.1*, *NRT2.1* and *ANR1* were upregulated by BES1.

BES1 physically interacts with LBD37 and represses its binding ability to GCGGCG consensus motif (**Figures 5**, **6**). It is likely that BES1 regulates the function of LBD37/38/39 at multiple levels. On the one hand, BES1 can directly bind to the E-Box and BRRE of LBD37/38/39 promoters and suppress these genes expression (**Figures 3**, **4**). On the other hand, BES1 can interact with LBD37/38/39 and inhibit LBD37 binding to its targets (**Figures 5**, **6**). Direct interaction between BES1 and its respective target transcriptional factors has been recognized as a general regulatory mechanism by which plant integrates BR signaling pathway with other pathways. For example, BES1 interacts with its target genes MYB30, MYB12 and HAT1 to coordinate BR-responsive genes expression (Li et al., 2009; Ye et al., 2012; Zhang et al., 2014). Several drought-responsive transcriptional factors, such as RD26, WRKY46/54/70 and TINY were identified as BES1 target genes according the BR-responsive genes transcriptome and BES1 ChiP-chip sequence data (Yu et al., 2011; Chen et al., 2017; Ye et al., 2017; Xie et al., 2019). Recent studies revealed that BES1 not only directly binds to the promoters of these drought-responsive genes, but also regulates the function of these genes through interacting with them, which are important for the coordination of BR-regulated growth and drought responses (Kono and Yin, 2020). Likewise, previous ChIP-chip studies indicated that LBD37/38/39 were targets of BES1 and BZR1. LBD37/38/39 were identified as novel repressors of N availability, which regulate the inhibitory effect of N/NO_3_^-^ on anthocyanin biosynthesis and influence nitrogen-responsive genes, and further affect nitrogen Status, growth, and nitrogen-dependent shoot branching (Rubin et al., 2009). In this study, we reported that LBD37 and its two homologous are also the repressors of LN-induced foraging responses of root, as overexpression of LBD37 resulted in reduced elongation of lateral root in response to LN and several LN-responsive genes involved in LN-induced lateral growth and development were suppressed by LBD37 (**Figure 7**). BES1 and LBD37 have opposite effects in the regulation of LN-responsive genes, such as *NRT1.1*, *NRT2.1* and *ANR1*. When overexpressing LBD37 in *bes1-D* or *35S:BES1-GFP* background, LBD37-inhibited lateral root elongation and LN-responsive genes were recovered to Col-0 levels, while the lateral root elongation and LN-responsive genes in *BES1-RNAi* plants were almost insensitive to LN, indicating an inhibitory effect of BES1 on the function of LBD37. These findings expand our understanding of the functions of BRs in transcriptional regulation of nitrogen responses and provide additional insight into the mechanisms by which plants coordinate lateral root growth and development at changing N availability.

In summary, we demonstrated that BES1-LBD37 module acts as key node downstream of BR signaling and nitrate signaling to link these two pathways. Mild nitrate deficiency induces both transcription level and activity of BES1 and activated BES1 promotes LN response in lateral growth and development. In addition, BES1 can inhibit the inhibitory effects of three key repressors of nitrate signaling LBD37/38/39 on root foraging response to LN through directly binding to their promoters and forming complexes with them. In future work, it will be important to investigate how BR and nitrate signaling regulates the formation of BES1-LBD37 complex.

## Data availability statement

The sequence data for genes described in this article are publicly available. This data can be found here: The Arabidopsis Information Resource (TAIR) under the following accession numbers: BES1 (AT1G19350), LBD37 (AT5G67420), LBD38 (AT3G49940), LBD39 (AT4G37540), NRT1.1 (AT1G12110), NRT2.1 (AT1G08090), ANR1 (AT2G14210).

## Author contributions

WT and SC designed the research. SC and JC did the experiments with XY and QZ’s assistant and analyzed all the data. WT and SC wrote the manuscript together. WT, YY and VD were involved in the data discussions. VD and YY edited the manuscript. All authors contributed to the article and approved the submitted version.

## Funding

This research was funded by the Natural Science Foundation of China (32000217, U20A20179 and 31850410483); Sichuan innovation talent project in science and technology (2020JDRC0065). The Natural Science Foundation of Southwest University of Science and Technology (18ZX7131, 19zx7153).

## Acknowledgments

We thank Prof. Yanhai Yin (Iowa State University) for providing the *bes1-D*, *BES1-RNAi* seeds and BES1 antibody. We also would like to thank Prof. Jianmin Zhou (Institute of Genetics and Developmental Biology, Chinese Academy of Sciences) for providing the pCAMBIA-1300-nLuc and pCAMBIA-1300-cLuc vectors.

## Conflict of interest

The authors declare that the research was conducted in the absence of any commercial or financial relationships that could be construed as a potential conflict of interest.

## Publisher’s note

All claims expressed in this article are solely those of the authors and do not necessarily represent those of their affiliated organizations, or those of the publisher, the editors and the reviewers. Any product that may be evaluated in this article, or claim that may be made by its manufacturer, is not guaranteed or endorsed by the publisher.
