# Supplementary material for: Interaction of BES1 and LBD37 transcription factors modulates brassinosteroid-regulated root forging response under low nitrogen in arabidopsis
Source: Front Plant Sci. 2022 Sep 28;13:998961. doi: 10.3389/fpls.2022.998961 (PMC9555238; doi:10.3389/fpls.2022.998961)
Supplement: Supplementary file 1 [file DataSheet_1.pdf]

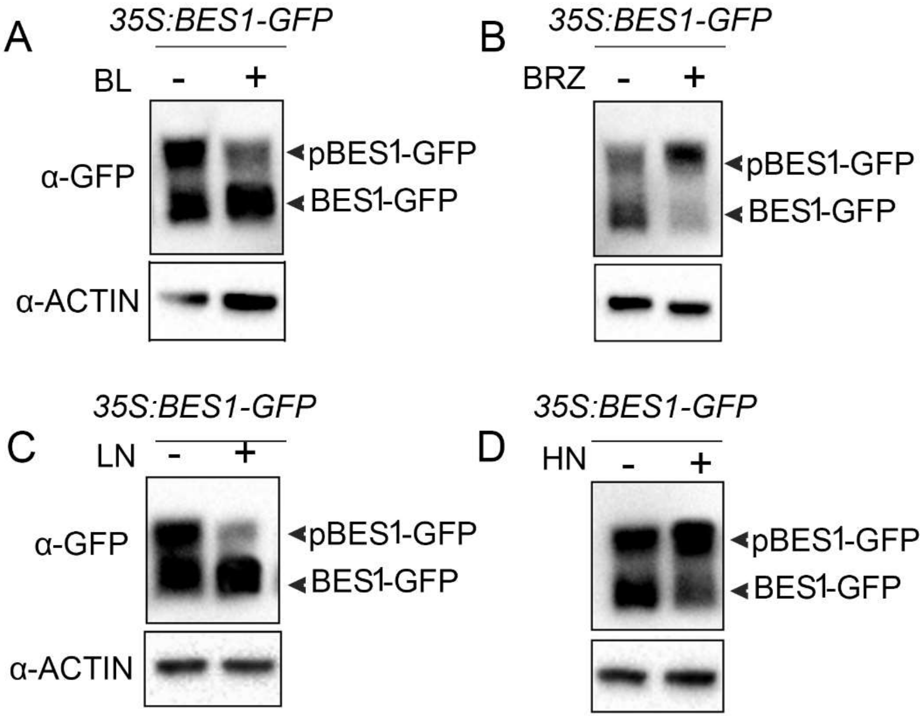

Supplementary Figure S1 | Immunoblotting showing phosphorylation status of BES1 in the seedlings used for ChIP-qPCR assay in Figure 4E and 4F. 14-day-old Col-0 and *35S:BES1-GFP* seedlings with BL (**A**), BRZ (**B**), LN (**C**) or HN (**D**) treatment were used to detect BES1-GFP protein using immunoblot with anti-GFP antibody.

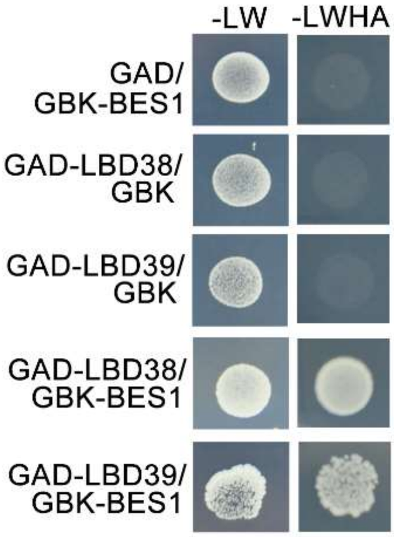

Supplementary Figure S2 | Interaction between BES1 and LBD38/39. The GAL4 activation domain expressed by pGADT7 (shown as GAD) and the GAL4 binding domain expressed by pGBKT7 (shown as GBK) were used as negative controls. The experiment was repeated three times with similar results, and representative images are displayed.

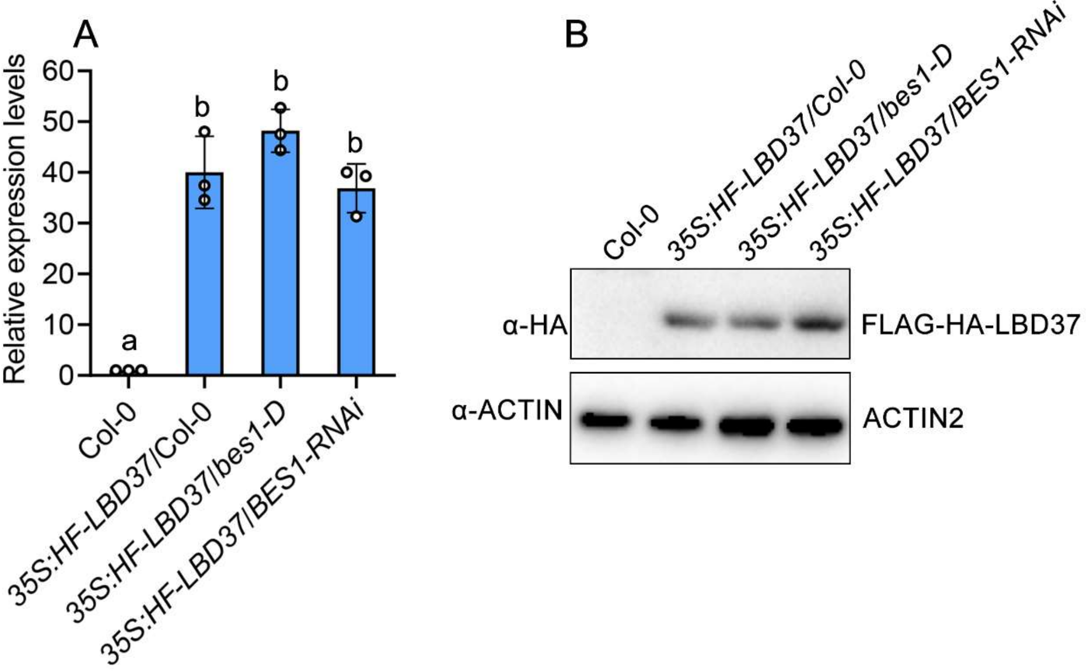

Supplementary Figure S3 | Transcript (A) and protein (B) levels of LBD37 in wild-type (Col-0), *35S:FLAG-HA-LBD37*/Col-0, *35S:FLAG-HA-LBD37/bes1-D* and 35*S:FLAG-HA-LBD37/ BES1-RNAi*.

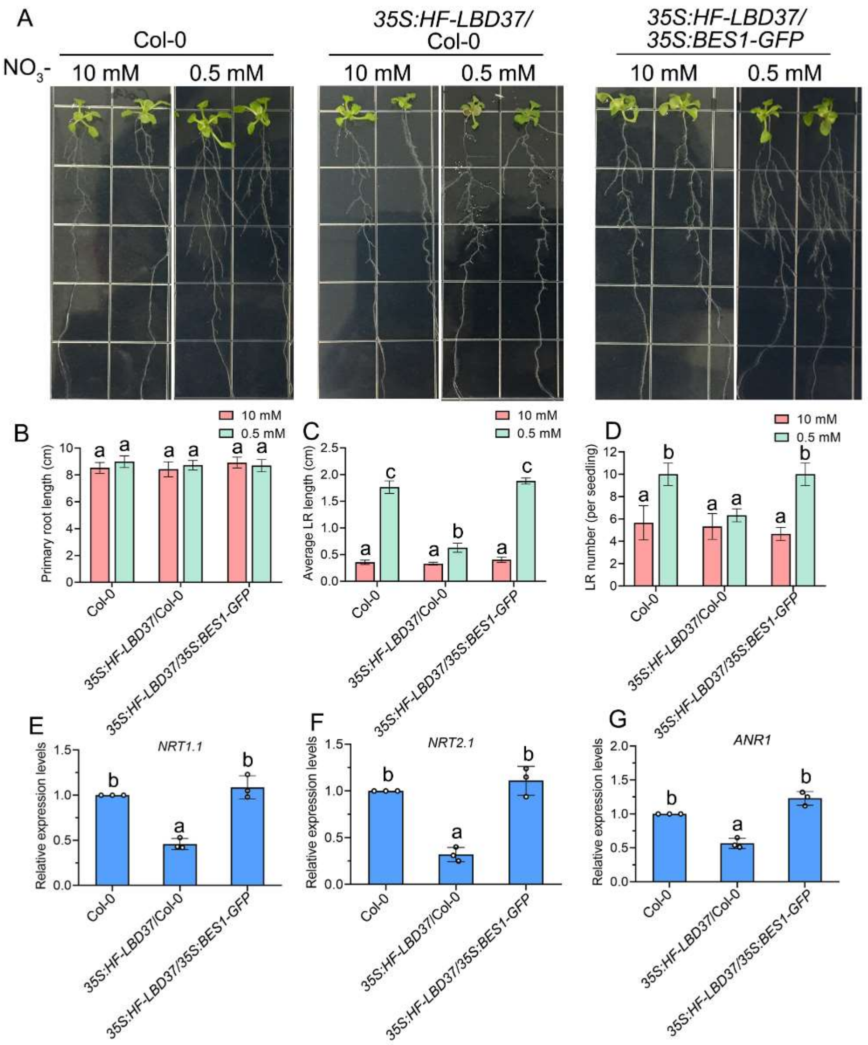

Supplementary Figure S4 | The LN-responsive phenotypes of wild-type (Col-0), *35S:HF-LBD37/*Col-0 and *35S:HF-LBD37/35S:BES1-GFP*. 5-day-old seedlings were precultured on medium containing 10 mM NO_3^- and then transferred to HN medium or LN medium for another 9 days for quantification of primary root length (B), average lateral root (LR) length (C), and lateral root (LR) number (D). Error bars indicate the s.d. (n=10-15). Different Lowercase letters above the bars indicate statistically significant differences between samples ($p < 0.05$, two-way ANOVA). (E-F) Expression analysis of LN-responsive genes *NRT1.1* (E), *NRT2.1* (F) and *ANR1* (G) in the roots of 7-day-old (Col-0), *35S:HF-LBD37/*Col-0 and *35S:HF-LBD37/35S:BES1-GFP* seedlings at LN. The expression levels of genes in the Col-0 seedlings were set to 1.00. Error bars represent s.d. of three biological repeats. Different Lowercase letters above the bars indicate statistically significant differences between samples ($p < 0.05$, one-way ANOVA).
